# Staged treatment using external fixation for comminuted fracture of first metatarsal: a case report

**DOI:** 10.1093/jscr/rjaf889

**Published:** 2025-11-11

**Authors:** Ryogo Furuhata, Yusuke Shiba, Atsushi Tanji

**Affiliations:** Department of Orthopaedic Surgery, Ashikaga Red Cross Hospital, Ashikaga-shi, Tochigi 326-0843, Japan; Department of Orthopaedic Surgery, Ashikaga Red Cross Hospital, Ashikaga-shi, Tochigi 326-0843, Japan; Department of Orthopaedic Surgery, Ashikaga Red Cross Hospital, Ashikaga-shi, Tochigi 326-0843, Japan

**Keywords:** first-metatarsal fracture, external fixation, plate, staged treatment, case report

## Abstract

In high-energy midfoot fractures with severe soft-tissue damage, immediate osteosynthesis is often challenging owing to the lack of subcutaneous tissue and forefoot swelling. We herein report a case of comminuted first-metatarsal fracture with marked shortening and poor skin condition, managed using a staged approach with external fixation. A 52-year-old man presented with left forefoot pain after a tiller-related mishap. Computed tomography revealed a comminuted first-metatarsal fracture with shortening. Owing to poor condition of the skin, a staged treatment was adopted. In the first stage, an external fixator was applied to correct the metatarsal shortening; in the second stage, a fusion plate was placed between the first metatarsal and medial cuneiform. The external fixator was removed after 4 weeks. The patient achieved bone union with satisfactory functional outcomes. External fixation allows for delayed definitive fixation until tissue physiology improves, thereby enhancing the stability of internal fixation.

## Introduction

First-metatarsal fractures are rare, accounting for 1.5% of all adult fractures [1], and are commonly caused by standing falls or direct blows [2]. Cases with significant instability and displacement require surgical intervention [3]. Surgical treatment for first-metatarsal fractures includes smooth-wire, screw, and plate fixations [3]. However, immediate open reduction and internal fixation using plates and screws is often challenging owing to the absence of subcutaneous tissue and forefoot swelling [4]. Moreover, delayed or inadequate reduction for severe midfoot fractures has been associated with poor outcomes and further complications [5, 6].

Herein, we report a case of a comminuted fracture of the first metatarsal with marked shortening and poor skin condition, managed using a staged treatment with external fixation.

## Case report

**Figure 1:**
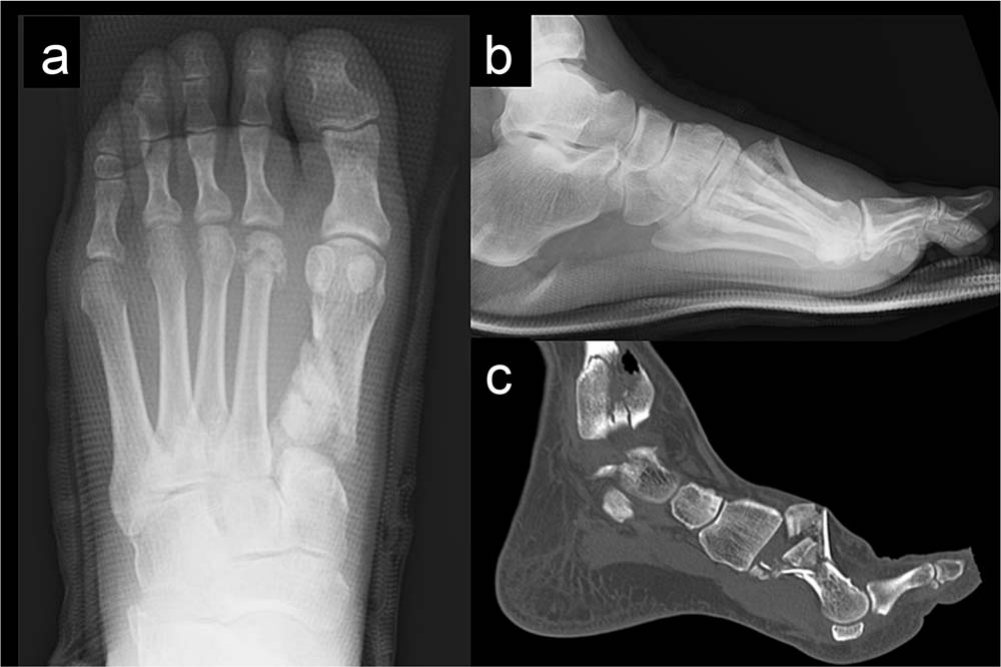
Radiography (a, b) and computed tomography (c) images of the left forefoot showing the comminuted first-metatarsal fracture.

A 52-year-old man presented to our hospital with pain in the left ankle and forefoot after his left foot was caught in a wall while operating a tiller. Deformity was observed in the left ankle joint and the left forefoot. No evidence of neurovascular injuries or wounds was observed. The patient had a history of diabetes and hypertension. Radiography showed fractures of the left first metatarsal (Fig. 1a and b), tibial pylon, and distal fibula. Computed tomography (CT) revealed that the base of the first metatarsal was comminuted (Fig. 1c), and a dorsal skin prominence was identified due to a displaced dorsal fragment.

**Figure 2:**
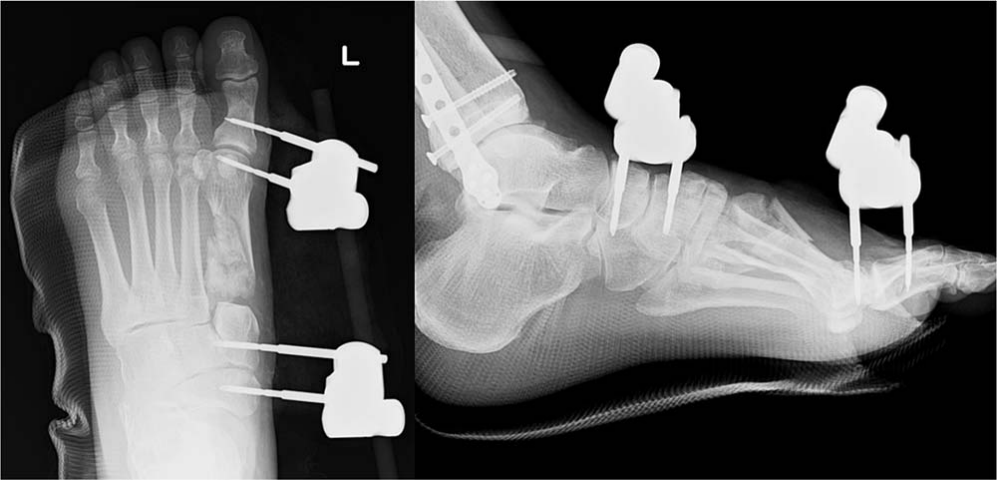
Radiography images immediately after the first surgery.

**Figure 3:**
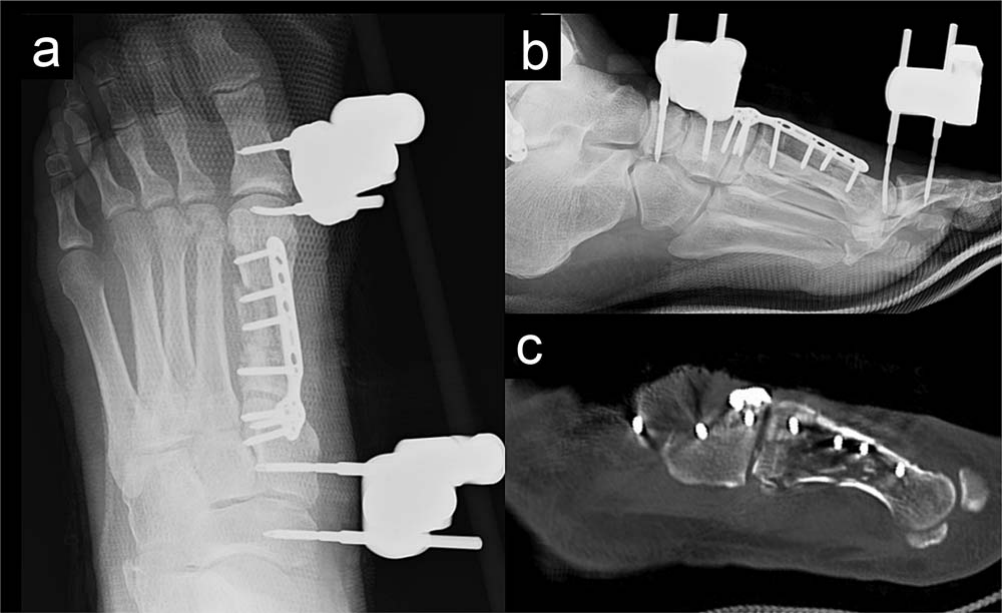
Radiography (a, b) and computed tomography (c) images immediately after the second surgery.

We performed surgery in two stages, owing to the poor condition of the skin. In the first stage, we performed screw fixation for the tibial pilon fracture and plate fixation for the distal fibular fracture. For the first-metatarsal fracture, half pins for external fixation were inserted into the first proximal phalanx, first metatarsal, medial cuneiform, and navicular bone. The external fixator (The Small External Fixation System, Synthes, Oberdorf, Switzerland) was stabilized with the first reduction in the metatarsal shortening deformity (Fig. 2). One week later, the second-stage surgery was performed after the swelling of soft tissues on the dorsum of the foot had improved. A longitudinal skin incision was made on the dorsum of the first metatarsal, and a fusion plate (Variable Angle LCP Forefoot/Midfoot system, Synthes) was placed on the dorsal aspect of the first metatarsal and the medial cuneiform. Three screws were inserted into the medial cuneiform, and four screws were inserted into the distal end of the first metatarsal. Postoperative radiography and CT images are shown in Fig. 3.

**Figure 4:**
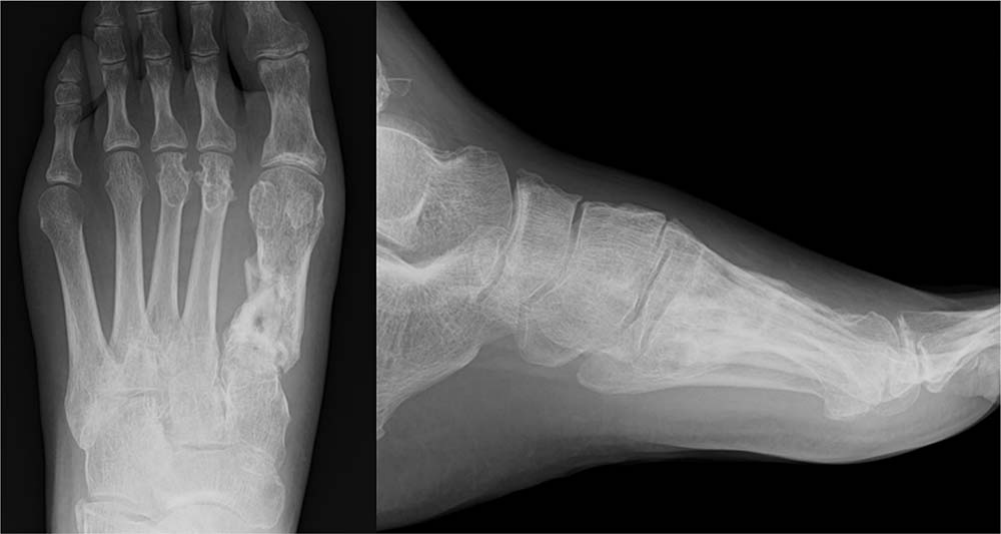
Radiography images 1 year after surgery.

The external fixator was removed 4 weeks after the first surgery. Full weight-bearing was allowed 6 weeks after the operation. The plate was removed after confirming bone union. The patient was able to return to agricultural work 6 months postoperatively, and the American Orthopedic Foot and Ankle Society score 1 year post-surgery was 84. The follow-up radiography image taken at this stage is shown in Fig. 4.

## Discussion

In this case, staged treatment using external fixation for a first-metatarsal fracture with marked shortening and comminution provided satisfactory radiological and functional outcomes.

To date, closed reduction and splinting have been widely used as a temporary measure for high-energy midfoot fractures wherein the soft tissue envelope is compromised and immediate open fixation is not feasible [7–9]. However, maintaining optimal alignment and adequate reduction with these temporary fixations is often challenging [7–9]. External fixation has been proposed as a potential solution to maintain alignment and stability in midfoot fractures while avoiding further devascularization [10, 11]. Additionally, external fixation allows for waiting until the soft tissue becomes amenable to definitive fixation [12]. Several clinical studies have reported that external fixation allowed delayed definitive fixation until local physiology is optimized and improves the final length and alignment of the fracture in high-energy midfoot fracture dislocations [12, 13].

The procedure used in this case has two additional advantages. First, while conventional procedures involve inserting external-fixation pins into the calcaneus and metatarsal bones [13, 14], this procedure involves inserting pins into the navicular bone, medial cuneiform, distal part of the first metatarsal, and first proximal phalanx, thereby allowing for a more specialized restoration of alignment and length of the first metatarsal. In cases with large bone defects in the first metatarsal, there have been a few reports of external fixation using a pin-insertion procedure similar to that in our case, which enabled recovery and maintenance of the length and alignment of the first metatarsal [4, 10]. Second, combining external and plate fixations until bone callus formation reduces the compression force at the fracture site and enhances the stability of the plate. In previous reports, the external fixation was removed when secondary open reduction and internal fixation or fusion were performed [12, 13]. The present case suggests that continuing external fixation until fracture stability is achieved may provide additional stability for internal fixation.

However, this procedure has potential disadvantages, including an increased risk of infection at the pin-insertion site and risk of vascular damage [15]. In the present case, to prevent vascular damage, we exposed the surface of bone directly and inserted half pins.

In conclusion, this case report provides insights into the treatment of comminuted first-metatarsal fractures with marked shortening. The use of external fixation allows for delayed internal fixation until the tissue physiology improves, which can enhance the stability of internal fixation. Further studies and case reports involving larger patient cohorts are needed to evaluate the postoperative effectiveness of this treatment for complex metatarsal fractures.

## Data Availability

Data supporting the findings of this study are available from the corresponding author upon request.
